# Cascading transitions toward unconventional charge density wave states in the quasi-two-dimensional monophosphate tungsten bronze P_4_W_16_O_56_

**DOI:** 10.1107/S2052252519016695

**Published:** 2020-01-16

**Authors:** Elen Duverger-Nédellec, Alain Pautrat, Kamil K. Kolincio, Laurence Hervé, Olivier Pérez

**Affiliations:** a CRISMAT, ENSICAEN/CNRS UMR6508, 6 Boulevard du Maréchal Juin, Caen, Normandie 14050, France; b Faculty of Applied Physics and Mathematics, Gdansk University of Technology, Narutowicza 11/12, Gdansk 80-233, Poland

**Keywords:** phase transitions, charge density waves, modulated structures, inorganic materials, aperiodic structures, X-ray diffraction, resistivity

## Abstract

Single crystals of the *m* = 8 member of the low-dimensional monophosphate tungsten bronzes (PO_2_)_4_(WO_3_)_2*m*_ family were grown by chemical vapour transport technique and the high crystalline quality obtained allowed a reinvestigation of the physical and structural properties. Resistivity measurements revealed three anomalies at *T*_C1_ = 258 K, *T*_C2_ = 245 K and *T*_C3_ = 140 K, never observed until now. Parallel X-ray diffraction investigations showed a specific signature associated with three structural transitions, *i.e.* the appearance of different sets of satellite reflections below *T*_C1_, *T*_C2_ and *T*_C3_. Several harmonics of intense satellite reflections were observed, reflecting the non-sinusoidal nature of the structural modulations and a strong electron–phonon coupling in the material. These transitions could be associated with the formation of three successive unconventional charge density wave states.

## Abstract 2

Successive transitions toward unconventional charge density wave states are shown by resistivity measurements and thermal X-ray diffraction.

## 1. Introduction   

Monophosphate tungsten bronzes with pentagonal tunnels (MPTBp) were discovered several decades ago during the quest for low-dimensionality conductors (Giroult *et al.*, 1981 ▸, 1982 ▸). Their structure is built from the regular intergrowth of PO_4_ tetrahedra slices and of ReO_3_-type slabs of corner-sharing octohedra WO_6_ giving rise to the formation of empty pentagonal tunnels at their junctions (Roussel *et al.*, 2001 ▸). PO_4_ slices have a thickness of one tetrahedron while the thickness of the ReO_3_-type slabs is directly related to an *m* parameter that ranges from 2 to 14. Consequently, the different members of the family have the chemical formula (PO_2_)_4_(WO_3_)_2*m*_. The PO_4_ slices provide electrons to the WO_3_ slabs; these slabs support the conductive character of the MPTBp.

The MPTBp is a unique system offering the possibility to tune the effective dimensionality via variation of the *m* parameter and thus thickness of the W–O block; the first term (*m* = 2) exhibits a 1D structural character with the formation of WO_3_ chains, the intermediate terms are quasi-2D, the largest terms (*m* ≥ 10) are 2D and for *m* = ∞ (WO_3_) the system becomes 3D. Moreover, although the number of 5*d* electrons per unit cell is constant and equal to 4 for the members of the MPTBp, the average conduction electron density per W atoms decreases with *m* and therefore with the dimensionality. One strong interest of MPTBs arises from this possibility to investigate, within one system, the impact of dimensionality on electronic instabilities such as superconductivity, spin-density waves and charge density waves (CDWs), essentially associated with 1D and 2D conductors (Schlenker, 1996 ▸; Greenblatt, 1996 ▸).

MPTBp has been the subject of numerous studies that have shown the existence of transitions toward CDW states [see Table 1 of Roussel *et al.* (2001 ▸) for an exhaustive overview]. The different authors report a Peierls-type transition characterized by the opening of a gap at the Fermi surface and, consequently, by a loss of charge carriers, accompanied by a periodic lattice distortion (Peierls, 1955 ▸). The CDW transition thus has a structural signature directly visible with X-ray diffraction (XRD) experiments with the appearance of satellite reflections on the XRD pattern below the transition temperatures related to a structural modulation. Monitoring the XRD pattern while varying the temperature enabled P. Foury, A. Ottolenghi and J.-P. Pouget to determine the transition temperatures and the positions of the satellite reflections for each term of the MPTBp family (Ottolenghi & Pouget, 1996 ▸; Foury & Pouget, 1993 ▸). Additionally, the corresponding anomalies in the electronic transport properties have been predicted and reported in the literature for the low terms of the series, *i.e.* for *m* ≤ 6 (Wang *et al.*, 1989 ▸; Schlenker *et al.*, 1996 ▸; Hess *et al.*, 1996 ▸; Kolincio *et al.*, 2016 ▸; Kolincio, 2013 ▸).

For *m* ≥ 7 the electronic transport shows a bad metal behaviour dominated by electron–electron interactions and weak localization effects, which is in contrast with the metallic behaviour observed for low members (Kolincio, 2013 ▸; Rötger *et al.*, 1994 ▸; Hess *et al.*, 1997*a* ▸,*b* ▸; Dumas *et al.*, 2000 ▸). This border between high and low terms is also detected by XRD experiments with the observation at the transition of commensurate modulated structures characterized by intense satellite reflections associated, at least for the *m* = 10 member, with an anti-ferroelectric order for the W atoms (Roussel *et al.*, 2000 ▸).

**Figure 1:**
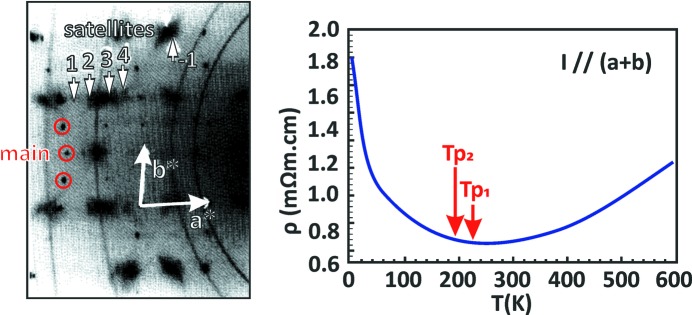
Previous studies for P_4_W_16_O_56_: XRD pattern obtained by Ottolenghi *et al.* (1995 ▸) at 37 K and resistivity measurements published by Dumas *et al.* (2000 ▸). Changes have been applied to the original figure; red circles show the positions of the main reflections and some satellite reflections and axes are highlighted with more contrast.

The *m* = 8 member with the chemical formula P_4_W_16_O_56_, as already mentioned in the works by Ottolenghi *et al.* (Ottolenghi & Pouget, 1996 ▸; Ottolenghi *et al.*, 1995 ▸), occupies a very specific place in the MPTBp phase diagram at the borderline between low- and high-*m* type behaviour. This compound has been the subject of numerous studies from both structural properties and physical points of view (Ottolenghi & Pouget, 1996 ▸; Schlenker *et al.*, 1996 ▸; Hess *et al.*, 1997*a* ▸; Dumas *et al.*, 1999 ▸, 2000 ▸; Ottolenghi *et al.*, 1995 ▸; Labbé *et al.*, 1986 ▸; Witkowski *et al.*, 1997 ▸; Domengès *et al.*, 1984 ▸). Its atomic structure in the fundamental state is well known (Labbé *et al.*, 1986 ▸). The thermo-diffraction studies performed in the works by Ottolenghi *et al.* (Ottolenghi & Pouget, 1996 ▸; Ottolenghi *et al.*, 1995 ▸) by cooling down to *T* = 35 K reveal the occurrence of successive sets of broad satellite reflections located on diffuse lines or sheets (see Fig. 1 ▸). The authors identified three different sets of satellites observed below *T*_C0_ = 250 K, *T*_C1_ = 220 K and *T*_C2_ = 200 K characterized by three wavevectors **q**_0_ = [0.27 (3), 0, 0], **q**_1_ = [0.47 (2), 0.02 (1), 0.15 (10)] and **q**_2_ = [0.19 (2), 0.03 (1), 0.06 (3)]. The observation of the three sets of broad satellite reflections is in agreement with the setup of short-range-order structural modulations. Additionally, at all temperatures, diffuse lines perpendicular to the **a** ± **b** directions are reported; the authors claim these diffuse lines are a signature of quasi-1D CDW instabilities already observed in the lower *m* members. The analysis of the resistivity versus *T*, from 3 to 600 K, for *m* = 8, reported by Dumas *et al.* (2000 ▸), has not revealed any anomalies (see Fig. 1 ▸). Ottolenghi *et al.* propose that both of the instabilities prevent the development of a long-range-ordered (LRO) CDW (Ottolenghi & Pouget, 1996 ▸). These results are very surprising with regards to the resistivity measurements carried out on the terms *m* = 7 and *m* = 9, where clear, yet unconventional, signature for a CDW state was observed.

Since all these measurements are highly dependent on the quality of the samples, we present in this article a reinvestigation of the *m* = 8 member from both physical properties and crystallographic points of view, yet this time performed with newly synthetized high-quality crystals.

## 2. Experimental   

### 2.1. Synthesis   

Dark pink–purple single crystals, platelet-like with a surface parallel to the (**ab**) plane, were obtained by chemical vapour transport technique described by Roussel *et al.* (1996 ▸). To further improve the crystals quality, particular attention has been paid to the applied thermal gradient as well as to the proportion of precursors introduced into quartz-sealed tubes in order to control the internal pressure inside the tubes. The crystalline quality and the orientation of the crystals were systematically controlled at room temperature by XRD measurements. Samples with appropriate size for physical and structural characterizations were collected from the same batch; one order of magnitude is usually observed in the sample dimension used for these two techniques.

### 2.2. Resistivity measurements   

Samples with large dimensions were chosen for the resistivity measurements. However, the preliminary XRD experiments were performed at room temperature; only single crystals exhibiting sharp Bragg peaks, *i.e.* with the lowest stacking faults rate, were chosen for further analysis. Qualified specimens were investigated by XRD at 100 K. Three different types of sample can be identified from the observation at 100 K: crystals with occurrence of additional sharp satellite reflections (SR), crystals exhibiting diffuse scattering (DS) located in the (a*, b*) planes and crystals without any additional diffraction features (NO); SR crystals are considered to present long-range-order states while DS crystals exhibit only short-range-order states and NO crystals show no additional order. Samples from each of these different categories (called SR, DS and NO in the following) (see Section ‘Thermal XRD: Screening’[Sec sec3.2.1]) were selected. The different samples were cleaned in acetone for five minutes then in HF (5%) at 70°C for one hour (Hess *et al.*, 1997*c* ▸). Four pads of platinum were deposited at the surface of the crystal (**ab** plane) using sputtering. Then one gold wire (25 µm in diameter) was glued onto each pad with ep­oxy silver paste (Dupont 6828). For technical reasons, we used a four probe-like geometry, where current non-uniformity can occur along the thickness in an anisotropic conductor and the measured resistivity can not be directly associated to ρ_ab_. Note however that because of the reduced thickness of the sample compared with the square root of the surface area (a factor of 3), the difference between the effective thickness probed by the current and the real thickness of the sample should be reduced. None of the measured resistivity versus temperature [*R*(*T*)] curves show signs of insulating/semiconducting components which would indicate **c**-axis resistivity components, so we can assume that the reported resistivity behaviour is related to the **ab** conducting plane.

**Figure 2:**
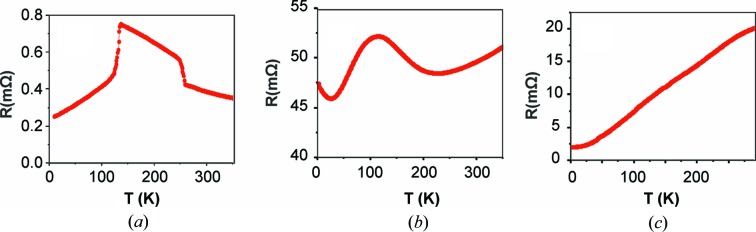
Resistance temperature dependence for crystals of P_4_W_16_O_56_ for categories SR (*a*), DS (*b*) and NO (*c*).

Resistivity measurements were performed using a physical property measurement system (PPMS) Quantum Design. Resistance data were obtained during cooling of the sample in the 300–2 K temperature range using an alternating 5 mA current at 7 Hz injected along the (**ab**) crystallographic plane. Three different behaviours can be identified for the three categories of samples; they are reported in Fig. 2 ▸. Crystals of category SR show two strong discontinuities, crystals of DS exhibit metallic behaviour with a large resistivity bump, and crystals of NO have a purely metallic behaviour without any incident. Only crystals of the SR category shall be considered to be of sufficient crystalline quality and hence representative of the intrinsic properties of *m* = 8. Future studies will be conducted on samples of this category.

### 2.3. XRD measurements   

Crystals with less than 50 micrometres in the largest dimension exhibiting sharp Bragg peaks and pre-transitional diffuse scattering were then used to perform temperature-dependant monitoring of the XRD pattern. The data were collected using two different instruments: (I) an automatic 4-circles Bruker–Nonius diffractometer equipped with an *APEX2* CCD detector and a molybdenum Incoatec microfocus source, and (II) a Synergy-S Rigaku 4-circles diffractometer equipped with a Hypix 6000 hybrid photon-counting detector and Mo PhotonJet-S microfocus X-ray sources. An Oxford cryostream 700 system was used to cool down the crystal with a ramp of 5 K min^−1^. A thermalization time of 5 min was applied before each measurement. The collection strategy was performed with *APEX2* (Bruker, 2012 ▸) or *CrysAlisPro* (Agilent, 2014 ▸) software depending on the diffractometer used, and the data analysis was carried out using *CrysAlisPro* (Agilent, 2014 ▸).

## 3. Results and discussion   

### 3.1. Physical properties   

**Figure 3:**
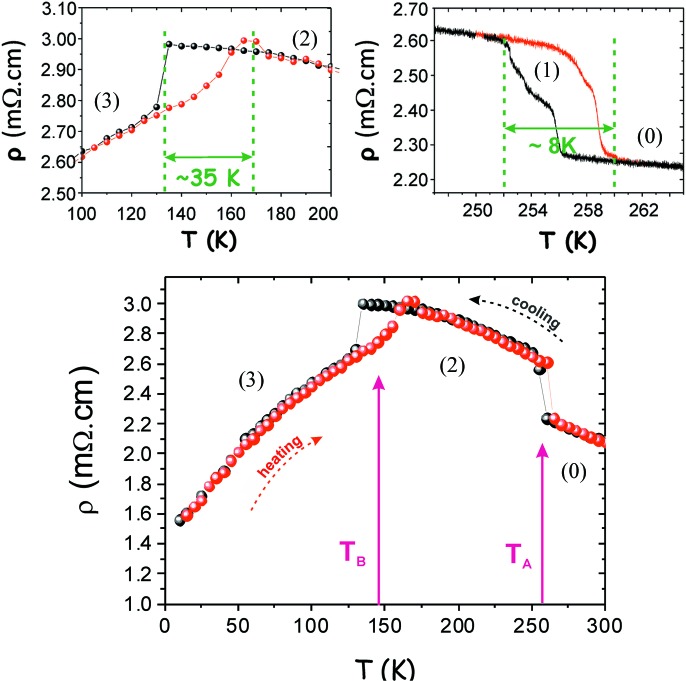
Resistivity versus temperature for P_4_W_16_O_56_. Red and black curves represent the evolution of the resistivity on heating and on cooling, respectively. Two enlargements of the regions around the temperature of the abrupt resistivity changes are shown (top left and top right). The (0), (1), (2) and (3) notations are associated with the states identified in Section 3.2.

The resistivity curves obtained for the P_4_W_16_O_56_ sample of category SR (approximate size of 550 × 250 × 150 µm) are shown in Fig. 3 ▸. The value of the resistivity is ∼2 mΩ cm at room temperature. This value is of the same order of magnitude as that reported by Dumas *et al.* (2000 ▸). Nevertheless, the temperature behaviour is very different: instead of a continuous resistivity variation, decreasing from 600 K to 250 K and then increasing down to the lowest temperatures, as measured by Dumas *et al.* (2000 ▸), two strong discontinuities are observed around *T*_A_ = 256 K and *T*_B_ = 135 K when cooling the sample. Details of these discontinuities are given in Fig. 3 ▸ and reveal their specificities; to improve the definition of the thermal hysteresis of resistivity between 245 and 265 K, the voltage was recorded continuously using a very small rate of temperature change (∼0.05 K min^−1^). The extensive study of the first discontinuity shows an 8 K thermal hysteresis and an intermediate step at 255 K (258 K), when the crystal is cooled (heated). The low-temperature anomaly is characterized by a large (35 K) and asymmetric hysteresis. These observations are then in favour of the final occurrence in the temperature dependence of the electrical resistivity of three different anomalies: the first one is observed at 255 K, the second one at 252 K (see detail of Fig. 3 ▸, top right) and the third one at 135 K on cooling (see detail of Fig. 3 ▸, top left).

### 3.2. Thermal XRD   

#### 3.2.1. Screenings   

Fine screenings of the diffraction pattern of high-quality single crystals of *m* = 8 were performed at various temperatures around the temperatures of the discontinuities observed in resistivity. Two different intervals of temperature were probed: (I) 220 K ≤ *T* ≤ 350 K to follow the first anomaly of Fig. 3 ▸ and (II) 100 K ≤ *T* ≤ 145 K for the second anomaly. The same region of the reciprocal space has been measured for these two temperature intervals; (*hkl*)* planes were reconstructed using *CrysAlisPro* software (Agilent, 2014 ▸).

**Figure 4:**
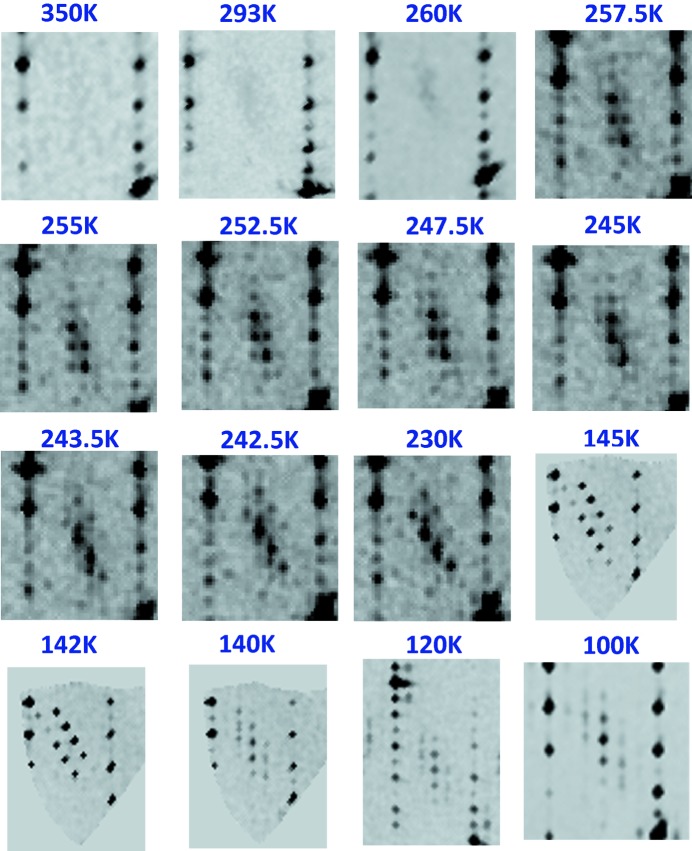
Thermal XRD: the same region of the (*h0l*)* planes has been measured at different temperatures on cooling.

The results obtained for the first interval are shown in Fig. 4 ▸. At 350 K, the diffraction pattern shows only reflections related to the unit cell *a* = 5.2943 (5) Å, *b* = 6.5534 (4) Å, *c* = 29.700 (4) Å, α = β = γ = 90° and is in perfect agreement with the structure already published in the work by Labbé *et al.* (1986 ▸). Around room temperature and down to about 260 K, pre-transitional diffuse scattering can be shown between the [*h00*]* rows. This diffuse signal condenses into sharp satellite reflections in the ∼258–247 K interval, signaling a transition towards a new structural state. Below 245 K, the satellites broaden and the position and intensity of the reflections observed between the [*h00*]* rows are modified step by step. This new scheme is stable from about 230 to ∼142 K and is characteristic of a new state. Finally, below ∼140 K the previous scheme is substituted by a new one, stable at least down to 80 K.

The previous screening then identifies four different structural states: state (0) for 260 K ≤ *T* ≤ room temperature, state (1) for 247 K ≤ *T*_C1_ ≤ 258 K, state (2) for 142 K ≤ *T*_C2_ ≤ 245 K and state (3) for *T*_C3_ ≤ 140 K

These different states can be attributed to the anomalies shown in the resistivity curves (Fig. 3 ▸). States (1) and (2) are associated with the signals observed at *T*_A_ with the double resistivity steps while state (3) corresponds to the anomaly at *T*_B_.

For the low-*m* members, successive transitions were already observed but each new structural state was characterized by the occurrence on the reciprocal space of an additional set of satellite reflections. Surprisingly, for the *m* = 8 member, after each transition the satellite reflections related to the previous state abruptly vanished in favour of the installation of a new set of satellite reflections. This specific behaviour could be attributed to a competition regime between different modulated states. Such phenomena were never observed in the MPTB family.

#### 3.2.2. Analysis of the different states   

The analysis of the different states shown above is important to obtain clues on the nature of the transitions. Now, let us determine the main characteristics of the different states observed for P_4_W_16_O_56_.

In state (0), which is the fundamental state, all the reflections are indexed using the orthorhombic cell given by *a* = 5.2821 (2) Å, *b* = 6.5341 (3) Å, *c* = 29.630 (2) Å. A structural refinement using the space group *P*2_1_2_1_2_1_ confirms that the crystal is in its fundamental orthorhombic state as already described by Labbé *et al.* (1986 ▸).

**Figure 5:**
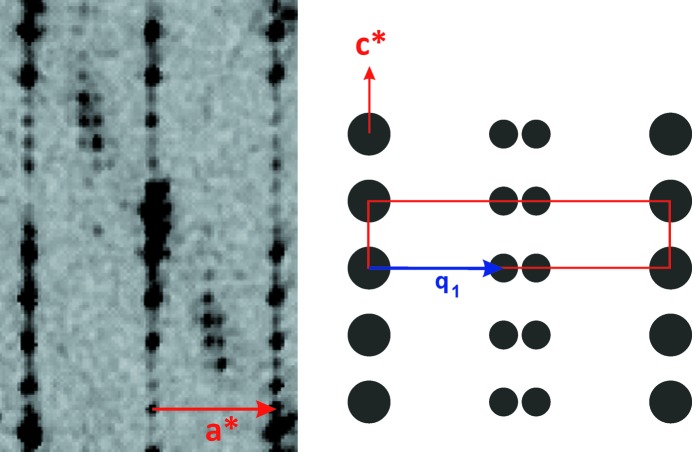
(*h0l*)* plane observed in state (1) at 247 K with a scheme explaining the indexing; the unit cell is drawn in red. The measurement was performed on the Bruker–Nonius diffractometer described in the Experimental Section[Sec sec2].

In state (1) (see Fig. 5 ▸) there is no evidence for cell distortion. The satellite reflections can be indexed by the modulation vector **q**_1_ = 0.446 (5) a*; the deviation from the rational value (4/9) of the component along a* is not significant and then the wavevector is considered to be in agreement with the setup of a commensurate modulated structure. The clear absence of a component along c* for the modulation vector of state (1) is in agreement with a distinct structural phase and not the coexistence of state (0) and state (2) (see below, the description of the diffraction pattern).

**Figure 6:**
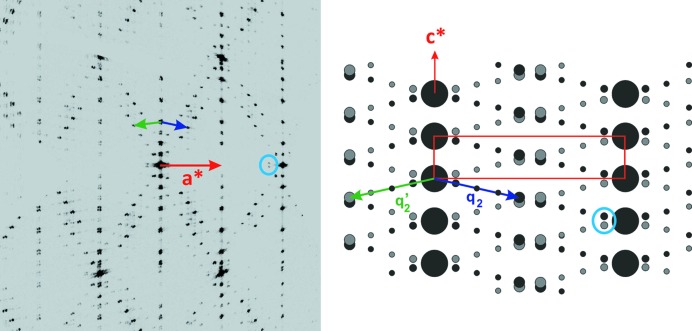
(*h0l*)* plane observed in state (2) at 200 K with a scheme explaining the indexing; the unit cell is drawn in red, **q**_2_ and **q**′_2_ are the modulation vectors related to the twin domains (**a**, **b**, **c**) and (**a**′, **b**′, **c**′), respectively. Cyan circles show the split second-order satellite reflections. The measurement was performed on the Synergy-S Rigaku diffractometer described in the Experimental Section[Sec sec2].

In state (2) (see Fig. 6 ▸), there is evidence for a distortion of the unit cell. The new cell *a* = 5.2998 (8) Å, *b* = 6.5591 (8) Å, *c* = 29.74 (4) Å and α = 90°, β = 90.221 (13)°, γ = 90° is compatible with a monoclinic symmetry with **b** as the binary axis. The indexing of the satellite reflections is more complex. They require the introduction of two wavevectors **q**_2_ and **q**_2_′ drawn in Fig. 6 ▸; satellite reflections are observed up to the fourth order. These two wavevectors are related by a twofold axis parallel to **c** and can be considered as characteristic of two twin domains of a non-merohedral twinning. This assumption of twinning can be confirmed by the observation in Fig. 6 ▸ of a splitting of the second-order satellite reflections (cyan circles in Fig. 6 ▸). Twins domains (**a**, **b**, **c**) and (**a**′, **b**′, **c**′) related by the twofold axis parallel to **c** are characterized by **q**_2_ = 4/9a* − 4/9c* and **q**_2_′ = 4/9**a**′* − 4/9**c**′* respectively; they have only rational components and are characteristic of a commensurate modulated structure. Each of these wavevectors is in agreement with the monoclinic symmetry (planar case).

All these observations demonstrate that the transition state (1) → state (2) is accompanied by a symmetry breaking from orthorhombic to monoclinic symmetry.

**Figure 7:**
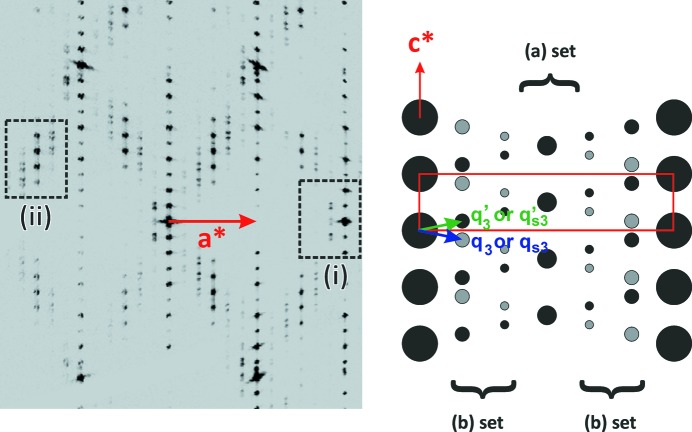
(*h0l*)* plane observed in state (3) at 100 K with a scheme explaining the indexing; the average unit cell is drawn in red, (**q**_3_ and **q**′_3_) and (**qs**_3_ and **qs**′_3_) are the modulation vectors proposed in the discussion of state (3) (see Table 1 ▸). The drawing was carried out using a radius, for each type of reflection, proportional to the structure factors. The measurement was performed on the Synergy-S Rigaku diffractometer described in the Experimental Section[Sec sec2].

In state (3) (see Fig. 7 ▸), a different distortion of the unit cell is shown: *a* = 5.3010 (2) Å, *b* = 6.5656 (2) Å, *c* = 29.761 (1) Å with α = β = 90° and γ = 90.25 (1)°. The metric is compatible with a monoclinic symmetry with **c** as the binary axis. An accurate observation of the diffracted intensities in the (*h0l*)* plane (Fig. 7 ▸) allows the identification of two independent sets of satellite reflections called (*a*) and (*b*) sets in the scheme of Fig. 7 ▸. Reflections of set (*a*) with positions defined by the vector 1/2a* − 1/2c* are intense and sharp. Reflections of set (*b*) are relatively weak and split along the c* direction; they can be indexed using two vectors related by a twofold axis parallel to **a** (1/6a* − 1/6c* and 1/6a* + 1/6c*); first- and second-order satellite reflections are identified. The observation of the weak but significant deviation of the angle γ from 90° induces a lowering of symmetry from Laue class of state (0) (*mmm*) to Laue class of state (3) (112/*m*) associated with the loss of twofold axes parallel to **a** and **b**. Following this observation and considering the splitting observed for reflections of the (*b*) set, *i.e.* the existence of both vectors 1/6a* − 1/6c* and 1/6a* + 1/6c*, it seems natural to consider the existence of two twin domains of a non-merohedral twinning related by a twofold axis parallel to **a**.

Two hypotheses can be proposed to index the diffraction pattern observed in state (3), they are related to the interpretation given for the reflections of the (*a*) set. (I) The reflections of the (*a*) set define a supercell (**a**_s_ = 2**a**, **b**_s_ = **b**, **c**_s_ = 2**c**) and can be considered as a set of main reflections. The reflections of the (*b*) set are then satellites of all the reflections of the supercell and are indexed with the wavevectors **qs**_3_ = 1/3a_s_* − 1/3c_s_* and **qs**′_3_ = 1/3a_s_* + 1/3c_s_*. This idea is supported by the observation of the equivalent distribution of the intensities of the reflections of the (*b*) set around the main reflections of the (**a**, **b**, **c**) cell and of the reflections of the (*a*) set [see for instance, areas (i) and (ii) in Fig. 7 ▸]. (II) The reflections of the (*a*) set are third-order satellites of the satellite reflections defined by the wavevectors **q**_3_ = 1/6a* − 1/6c* and **q**′_3_ = 1/6a* + 1/6c* already used to define reflections of the (*b*) set. As shown in Fig. 7 ▸, this interpretation leads to an unexpected feature: third-order satellites would have stronger intensity than first- or second-order satellites. However, the existence of the twin domains proposed above as well as a metric coincidence could explain this effect. Because of the weak deviation of the γ value from 90°, the proportion of the two twin domains can be considered as equivalent. Consequently, reflections of the (*b*) set, split over two positions, exhibit a one half-intensity in comparison with the intensity expected for a single domain crystal. On the contrary, the reflections of the (*a*) set corresponding to the summation of the intensity of {*h* a*, *k* b*, *l* c*, 3 × **q**′_3_}, {*h* a*, *k* b*, (*l* + 1)c*, 3 × **q**_3_}, {(*h* + 1)a*, *k* b*, *l* c*,−3 × **q**_3_} and {(*h* + 1)a*, *k* b*, (*l* + 1)c*,−3 × **q**′_3_} perfectly overlap.

Whatever the interpretation chosen, state (3) is characterized by a commensurate modulated structure. These two hypotheses will have to be tested during a structural resolution but the report of a wavevector 1/2a* + 1/2b* for different high-*m* members [Ottolenghi & Pouget (1996 ▸) for *m* = 12 and 14] is in favour of the first hypothesis. At which point, a discussion must be opened on the dimension of the modulation. The unit cell identified for state (3) shows a distortion with a metric compatible with a monoclinic symmetry with **c** as the unique axis. But the observation of the modulation vector (**q**_3_ or **qs**_3_) is fully in disagreement with the monoclinic symmetry for a (3 + 1)*d* modulated structure and this then implies a lowering of symmetry toward triclinic symmetry. In the assumption of a (3 + 1)*d* modulated structure the transition state (2) → state (3) would be accompanied by a symmetry breaking from monoclinic to triclinic symmetry; two additional twin domains are then expected. However, the possibility of a (3 + 2)*d* modulated structure with monoclinic symmetry cannot be fully rejected. In that case, **q**_3_ and **q**′_3_ (or **qs**_3_ and **qs**′_3_) would be related to the existence to a twofold axis parallel to **c** and not to the presence of twinning expected in the case of a first-order phase transition associated with such weak monoclinic distortion.

We can focus on the fact that the cell volume decreases linearly with the temperature when the symmetry of the system is conserved, and then a negative compressibility is observed at lower temperature with a variation of +1.09% when the system passes from state (1) to state (2) and a variation of +0.19% passing from state (2) to state (3). This can be explained by a rearrangement/rotation of the polyhedra constituting the crystal, increasing the symmetry of the structure when the temperature is increased (Miller *et al.*, 2009 ▸).

Note that although the transitions do not occur at the temperatures proposed by Ottolenghi *et al.* (Ottolenghi & Pouget, 1996 ▸; Ottolenghi *et al.*, 1995 ▸), the modulation vectors summarized in Table 1 ▸ have some similarities with those given by these authors. Thus, excluding the completely different components observed along c*, the components reported along a* are close to those we identify in the present work for **q**_2_ and **q**_3_. The quality of our diffraction patterns, with sharp satellite reflections, allows identification of the wavevectors with more accuracy and leads to the commensurate character of the modulations. Only crystal quality can explain such a difference.

### 3.3. Discussion   

The combination of X-ray thermo-diffraction and resistivity measurements performed on P_4_W_16_O_56_ reveals the existence of three discontinuous and hysteretic phase transitions (Fig. 3 ▸) towards three different states [states (1), (2) and (3)] characterized by commensurate modulated structures (see Table 1 ▸). It should be noted that the temperatures reported in diffraction and resistivity slightly differ for the second anomaly. However, the different procedures used for recording data (a very slow but continuous change of temperature for the resistivity and a thermalization before one hour measurements at each isotherm for the diffraction) may affect the exact position of transition. Moreover, the resistivity measurement is not a thermodynamic probe, in the sense that the anomaly in resistivity can coincide with a percolation-like process if the transition is somewhat heterogeneous at the sample scale (which is the case for hysteretic transition), and then anticipate the final temperature of complete transformation.

The observation of these three anomalies is very different from the behaviours reported in the literature for the low terms, *m* = 4 (Foury & Pouget, 1993 ▸; Hess *et al.*, 1996 ▸) and *m* = 6 (Foury & Pouget, 1993 ▸; Kolincio *et al.*, 2016 ▸; Foury *et al.*, 1991 ▸), for which classical continuous transitions toward CDW states with incommensurate modulations were shown. Note that all the satellite reflections observed for all the identified states are much more intense than those characteristic of CDW states for the low-*m* members of the MPTBp family. Thus, several orders of satellite reflections are observed for each modulated state. These phenomena, already observed for the high values of *m* and for *m* = 7 (Ottolenghi & Pouget, 1996 ▸; Foury & Pouget, 1993 ▸; Foury *et al.*, 1991 ▸), are coherent with strong electron–phonon coupling, which is unexpected for transitions toward classical CDW states. We will now discuss more accurately the results obtained for P_4_W_16_O_56_.

The transition toward state (3), characterized by a 35 K large asymmetric thermal hysteresis (Fig. 3 ▸, top left), also presents an unexpected bump of resistivity, visible between 160 and 175 K when the crystal is heated. Classically, a thermal hysteresis can be associated with a first-order transition and the associated phase coexistence due to superheating and undercooling processes. From a general viewpoint, it is expected to be symmetrical. An example for CDW transitions is the 1T–TaS_2_ case (Sipos *et al.*, 2008 ▸). Here, the presence of a very large and asymmetrical hysteresis implies that mechanisms other than purely thermodynamical should play a role. As an example, it is known that such large kinetic asymmetry can arise when electronic and structural degrees of freedom are strongly coupled, and when extended defects such as twins play a role in the nucleation of the phases (Fan *et al.*, 2011 ▸). It is worth noting that two hypotheses (summarized in Table 1 ▸) based on the existence of twin domains are proposed to describe the diffraction pattern of state (3) of P_4_W_16_O_56_. We speculate that the large asymmetric hysteresis observed at *T*_B_ is related to the presence of twin domains growing with temperature. In addition, when these domains grow and become in contact, a percolation path appears and could give birth to a abrupt change in the resistivity followed by a relaxation, explaining the bump seen in Fig. 3 ▸ (Efros & Shklovskii, 1976 ▸). This speculation is in favour of the hypotheses of a (3 + 1)*d* triclinic modulated structure.

**Figure 8:**
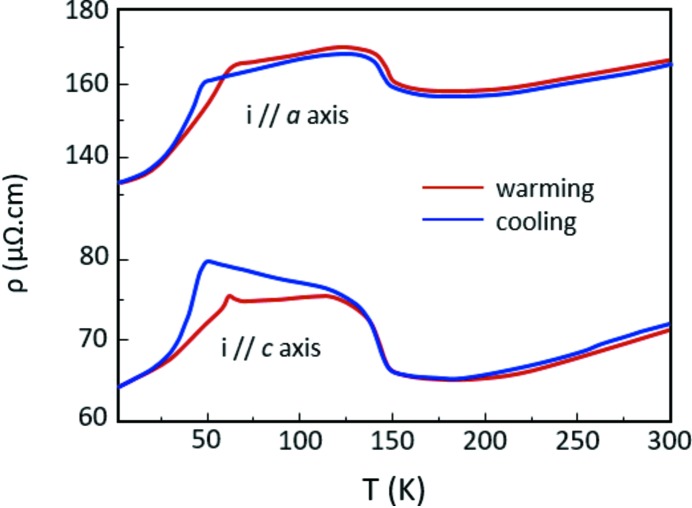
Resistivity versus temperature for Er_5_Ir_4_Si_10_ for current *i* // **a** and *i* // **c**. Blue and red curves represent the resistivity on cooling and heating, respectively. More details are provided in the references (Galli *et al.*, 2002 ▸; Ramakrishnan & Smaalen, 2017 ▸).

Additionally, the resistive behaviour observed for P_4_W_16_O_56_ resembles the one published in the work by Guyot *et al.* (1983 ▸) for a compound with structural similarities η-Mo_4_O_11_, which is well known to exhibit CDW states. However, a higher similarity exists with Er_5_Ir_4_Si_10_ (Galli *et al.*, 2000 ▸), which exhibits a quasi-identical resistive response (Fig. 8 ▸). The authors explain that the discontinuous transition at the lowest temperature is linked to a lock-in transition leading to an incommensurate CDW state–commensurate CDW state crossover. The first transition would establish a perfect nesting which is then destroyed by the second (last) one, enabling the material to return to a metallic state at low temperature. The last transition is also accompanied by a large hysteresis characterized by a broadening of the satellite reflections, due to the formation of domains (Galli *et al.*, 2002 ▸; Ramakrishnan & Smaalen, 2017 ▸). A bump in resistivity is also observed when the system is heated. Such similarities seem to confirm our hypothesis on the role of domains in the resistivity signature. But more interestingly, despite the original resistive behaviour of P_4_W_16_O_56_ in comparison with the low-*m* members of the MPTBp family, the analogy with η-Mo_4_O_11_ and Er_5_Ir_4_Si_10_ shows the possibility of matching the transitions observed for *m* = 8 with the formation of CDW states.

## 4. Conclusion   

In this article, we re-examined the intriguing structural and electronic properties of P_4_W_16_O_56_, the *m* = 8 term in the large family of monophosphate tungsten bronzes. This term was reported to be very peculiar since only short-range-ordered (SRO) modulated states with incommensurate wavevectors were observed, marking this material in the pretransitional regime of CDW condensation. There were no clear resistive anomalies, but a prominent weak localization component was reported. These puzzling results were tentatively attributed to the proximity in transition temperature of two different CDWs in competition and hence lacking correlation at long range. Here, we showed using thermal diffraction that the *m* = 8 MTBP can display LRO modulated states, and no evidence of weak localization phenomena is observed in the resistivity. It gives an experimental support for the proposed role of uncorrelated Peierls-like lattice distortions as an original source of disorder for weak localization (Dumas *et al.*, 2000 ▸).

The three LRO states have commensurate modulations and can be associated to three sharp and hysteretic resistivity changes. Then, P_4_W_16_O_56_ presents a very original behaviour with three commensurate states and symmetry-breaking transitions with first-order characteristics between each, going beyond the conventional mechanism of first-order transition between incommensurate and commensurate CDW (McMillan, 1976 ▸). These characteristics of unusual CDW transitions stand out from the low-*m* members where signatures of classical continuous Peierls transitions toward CDW state have been reported (Foury & Pouget, 1993 ▸; Foury *et al.*, 1991 ▸). The low-temperature 1/2a* − 1/2c* superstructure observed for state (3) can be viewed as due to the locking at 1/2 of the 4/9 components of the **q**_2_ wavevector shown for state (2). This locking to 1/2a* − 1/2c*, whose wavevector is also observed for *m* = 14 and 12, should be stabilized by the local establishment of the anti-ferroelectric (AFE) order of WO_3_ corresponding to the limit *m* infinite (Ottolenghi & Pouget, 1996 ▸). Such AFE order has been shown during the structure solution of the modulated structure characterizing the *m* = 10 member at room temperature (Roussel *et al.*, 2000 ▸). The setting up of AFE in the *m* = 8 member would outline the specific position of this MPTBp at the border in between the low- and high-*m* members.

Finally, it is worth noting that *m* = 8 crystals with apparent similar crystalline quality could present no modulation, SRO modulation or LRO modulation, with direct and major consequences to the electronic properties. We do not have a clear understanding of the genuine reason behind this contrasting behaviour that would deserve more detailed investigation such as microstructural probes, but we hope that it will help gain strong insights into the understanding of CDW stabilization.

## Figures and Tables

**Table 1:** Report of the different structural states showed by thermal XRD

State (0)	State (1)	State (2)	State (3)	
*T* ≥ 258 K	247 K ≤ *T* ≤ 258 K	142 K ≤ *T* ≤ 245 K	? ≤ *T* ≤ 140 K	
			Hypothesis 1	Hypothesis 2
*a* = 5.2943 (5) Å, *b* = 6.5534 (4) Å, *c* = 29.700 (4) Å, α = β = γ = 90°, *V* = 1030.46 Å^3^	*a* = 5.2821 (2) Å, *b* = 6.5341 (3) Å, *c* = 29.630 (2) Å, α = β = γ = 90°, *V* = 1022.64 Å^3^	*a* = 5.2998 (8) Å, *b* = 6.5591 (8) Å, *c* = 29.74 (4) Å, α = 90°, β = 90.221 (13)°, γ = 90°, *V* = 1033.81 Å^3^	*a*_s_ = 10.602 (4) Å, *b*_s_ = 6.5656 (2) Å, *c*_s_ = 59.522 (2) Å, α_s_ = β_s_ = 90°, γ_s_ = 90.25 (1)°, *V* = 4143.20 Å^3^	*a* = 5.3010 (2) Å, *b* = 6.5656 (2) Å, *c* = 29.761 (1) Å, α = β = 90°, γ = 90.25 (1)°, *V* = 1035.80 Å^3^
—	**q**_1_ = 0.446 (5)a* = 4/9a*	**q**_2_ = 0.444 (5)a* − 0.444 (5)c* = 4/9a* − 4/9c*	**qs**_3_ = 0.333 (5)a* − 0.333 (5)c* = 1/3a* − 1/3c*	**q**_3_ = 0.167 (5)a* − 0.167 (5)c* = 1/6a* − 1/6c*
Orthorhombic	Orthorhombic	Monoclinic	Triclinic	
—	—	2 twin domains	4 twin domains	
—	Commensurate modulation	Commensurate modulation	Commensurate modulation	
